# Evolution of computational models in BioModels Database and the Physiome Model Repository

**DOI:** 10.1186/s12918-018-0553-2

**Published:** 2018-04-12

**Authors:** Martin Scharm, Tom Gebhardt, Vasundra Touré, Andrea Bagnacani, Ali Salehzadeh-Yazdi, Olaf Wolkenhauer, Dagmar Waltemath

**Affiliations:** 1 Department of Systems Biology and Bioinformatics, University of Rostock, Rostock, 18051 Germany; 2 Department of Biology, Norwegian University of Science and Technology, Trondheim, 7491 Norway; 3 Stellenbosch Institute for Advanced Study, Wallenberg Research Centre at Stellenbosch University, Stellenbosch, 7600 South Africa

**Keywords:** BioModels, Physiome Model Repository, Model evolution, Model reuse, Difference detection

## Abstract

**Background:**

A useful model is one that is being (re)used. The development of a successful model does not finish with its publication. During reuse, models are being modified, i.e. expanded, corrected, and refined. Even small changes in the encoding of a model can, however, significantly affect its interpretation. Our motivation for the present study is to identify changes in models and make them transparent and traceable.

**Methods:**

We analysed 13734 models from BioModels Database and the Physiome Model Repository. For each model, we studied the frequencies and types of updates between its first and latest release. To demonstrate the impact of changes, we explored the history of a Repressilator model in BioModels Database.

**Results:**

We observed continuous updates in the majority of models. Surprisingly, even the early models are still being modified. We furthermore detected that many updates target annotations, which improves the information one can gain from models. To support the analysis of changes in model repositories we developed MoSt, an online tool for visualisations of changes in models. The scripts used to generate the data and figures for this study are available from GitHub github.com/binfalse/BiVeS-StatsGenerator and as a Docker image at hub.docker.com/r/binfalse/bives-statsgenerator. The website most.bio.informatik.uni-rostock.de provides interactive access to model versions and their evolutionary statistics.

**Conclusion:**

The reuse of models is still impeded by a lack of trust and documentation. A detailed and transparent documentation of all aspects of the model, including its provenance, will improve this situation. Knowledge about a model’s provenance can avoid the repetition of mistakes that others already faced. More insights are gained into how the system evolves from initial findings to a profound understanding. We argue that it is the responsibility of the maintainers of model repositories to offer transparent model provenance to their users.

## Background

The reuse of knowledge is key for the advancement of science [1]. Reusable computational models are provided by public repositories. Two major resources are BioModels Database [2] and the Physiome Model Repository [3]. Both repositories collect, curate, and publish models in standard formats, namely Systems Biology Markup Language (SBML) [4] and CellML [5]. When reusing a well documented model, researchers save time, effort, and money [6]. However, a lack of transparent documentation of the conditions and boundaries applied to the model, as well as a lack of provenance information, can lower the trust in a model. In contrast, a transparent communication of changes in models increases their value [7]. To build an informative history about a model, all its versions need to be publicly accessible, and all changes across versions have to be well described [8].

Both BioModels Database and the Physiome Model Repository provide versions of published models through their websites. Access to raw version information allows to further process the data and to study model changes. The BiVeS algorithm [9], for example, helps researchers to compute and analyse the differences between two versions of a model. Identified changes in model versions can then be classified using COMODI, an ontology of terms describing model changes [7].

In this paper, we analyse raw model versions with respect to the frequency and influence of changes. Using the BiVeS tool, we identify the changes between all released versions of models available from BioModels Database and the Physiome Model Repository. We identify update patterns, and we provide an example of a model’s history. The results show that models are indeed continuously subjected to changes. These changes, however, have different reasons, such as updates of the description format and error corrections. In order to provide interactive visualisations of changes in published models, we developed a freely available online platform.

## Methods

**Fig. 1:**
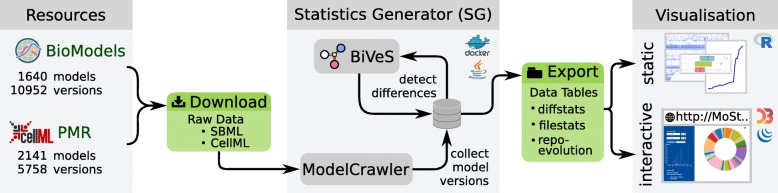
Pipeline used to obtain, analyse, and visualise the data. First, all relevant models and their versions are downloaded from BioModels and from the Physiome Model Repository (PMR) using the ModelCrawler. BiVeS detects and reports the differences between consecutive model versions. The Statistics Generator (SG) then exports the results as data tables, which collect statistics on models and changes. The data tables are used in R scripts and in the MoSt website to produce static and interactive visualisations, as presented in this paper

We generated the data presented and analysed in this paper following the schematic shown in Fig. 1. The heart of our pipeline is the Java tool *Statistics Generator*^1^ (SG), which wraps the *ModelCrawler*^2^ and *BiVeS* [9] to obtain and process the data. It first runs the ModelCrawler to retrieve all available model versions from *BioModels Database* and the *Physiome Model Repository*. The SG then uses BiVeS to calculate the differences between every subsequent version of each model. Afterwards, BiVeS’ output is evaluated and the results are stored in separate *data tables*. Based on these tables, a set of R scripts generates static figures, and the *ModelStats* (MoSt) website provides interactive visualisations of the data. The SG is available as a Docker image^3^. It can be used to regenerate the data tables. Our R^4^ scripts are available through the source code of the SG^5^. The MoSt website^6^ can be installed and run by everyone; the source code is available from GitHub^7^.

### The data

The data used to generate the figures originate from BioModels Database and the Physiome Model Repository. BioModels Database provides curated and non-curated models in SBML format. New models are submitted to the non-curated branch. Once the modelling results could be reproduced by a curator, the model moves to the curated branch. Release 31 of BioModels Database contains 640 models in the curated branch and 1000 models in the non-curated branch. For our study we considered all models in all release versions since the launch of the repository in April 2005 and the time of writing in July 2017 (10952 model versions).

The Physiome Model Repository provides curated and non-curated models, primarily in CellML format. The models are embedded in workspaces, which may contain further model related data, such as network visualisations, simulation descriptions, and links to previous versions of the studies. Particularly interesting and well documented revisions of workspaces can be published as exposures [10]. Workspaces may contain multiple models, which may be decomposed into different documents. For our study we treated every valid CellML document as a CellML model. In this work we retrieved all 2782 model files from 651 publicly available workspaces.

### ModelCrawler: acquiring models and versions

The ModelCrawler is a Java tool that retrieves models from open model repositories. It currently implements two modules: one for BioModels Database and one for the Physiome Model Repository. When retrieving data from BioModels Database, the ModelCrawler mirrors the corresponding FTP server at the EBI^8^, extracts the models of each release, and stores them locally. When retrieving data from the Physiome Model Repository, the ModelCrawler iterates through the list of public workspaces^9^, clones the corresponding GIT repositories, extracts the models in all revisions, and stores them locally.

In addition, the ModelCrawler collects and stores meta data, including information about the model’s origin and time stamps for each model version. For BioModels Database, the time stamps correspond to the release date of the database. The Physiome Model Repository provides precise version information through their repository backend (git-log) [10].

### BiVeS: comparing versions of a model

BiVeS compares the retrieved model versions and identifies the differences in the XML representation [9]. The tool distinguishes four types of changes (*insertion*, *deletion*, *move*, *update*) and three different kinds of entities in an XML document (*element node*, *attribute node*, *text node*) that are subjected to changes. BiVeS computes the differences between every two consecutive versions of each model retrieved by the ModelCrawler. In total, BiVeS generated 12467 deltas between model versions.

### Statistics Generator (SG): evaluating the BiVeS output

The results of BiVeS’ computation are post-processed and aggregated into three data tables.

The first table contains details about the models files in all available versions (*filestats*). Each row stores the number of (i) XML nodes, (ii) species, (iii) reactions, (iv) compartments, (v) functions, (vi) parameters, (vii) rules, (viii) events, (ix) units, (x) variables and (xi) components in the model. Additionally, information about the curation status of the model, encoding format, identifiers for model and version, and the URL to the model file is collected.

The second table contains data about the evolution of the repositories (*repo-evolution*). Starting from April 11th 2005 (BioModels emerged), it stores the number of models in BioModels and in the Physiome Model Repository. For each point in time, the details of the models, see (i)-(xi) above, are accumulated into three feature vectors. One vector for BioModels Database, one for Physiome Model Repository, and another one representing both repositories combined.

The third table contains details on the differences between two successive versions of a model (*diffstats*). Every version transition is examined with both the Unix *diff* tool (inserts and deletes) and BiVeS (inserts, deletes, updates, moves, and triggered operations [9]). Furthermore, each row in the table contains the corresponding model identifier and the identifiers for both versions of the model. Thus, every entry in the *diffstats* table can be linked to the model versions in the *filestats* table.

### Generating the static figures

**Fig. 2:**
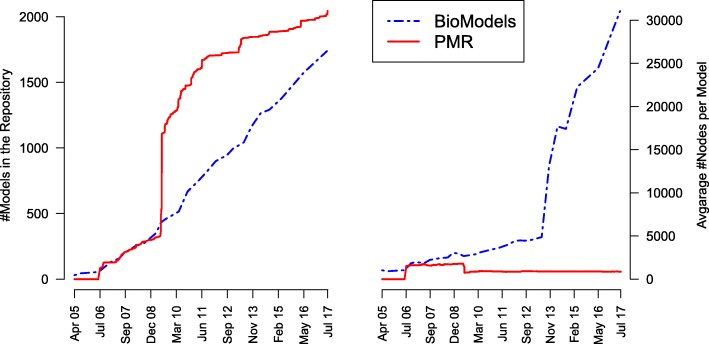
Number and size of models in BioModels Database and in the Physiome Model Repository. The plot shows the total number of models (left) and the mean number of nodes per model (right) in BioModels Database (dotted line) and in the Physiome Model Repository (solid line) since the launch of the databases until July 2017

**Fig. 3:**
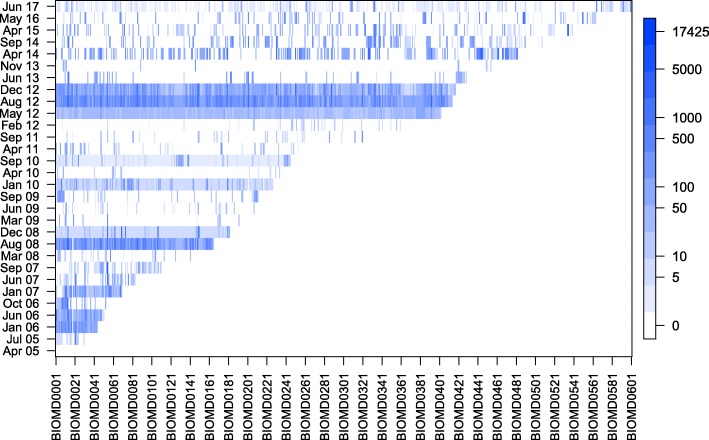
Updates of models in BioModels Database. The plot shows every recorded update of each model in the curated branch of BioModels Database. The rows (y-axis) show changes per official release of BioModels Database. The columns (x-axis) represent model files. Whenever a model was updated, a blue vertical line indicates how many changes BiVeS detected between the old and new version of the model. Dark blue indicates many changes (maximum of 17 425 operations), light blue indicates few changes (minimum of 0-5 change operations)

**Fig. 4:**
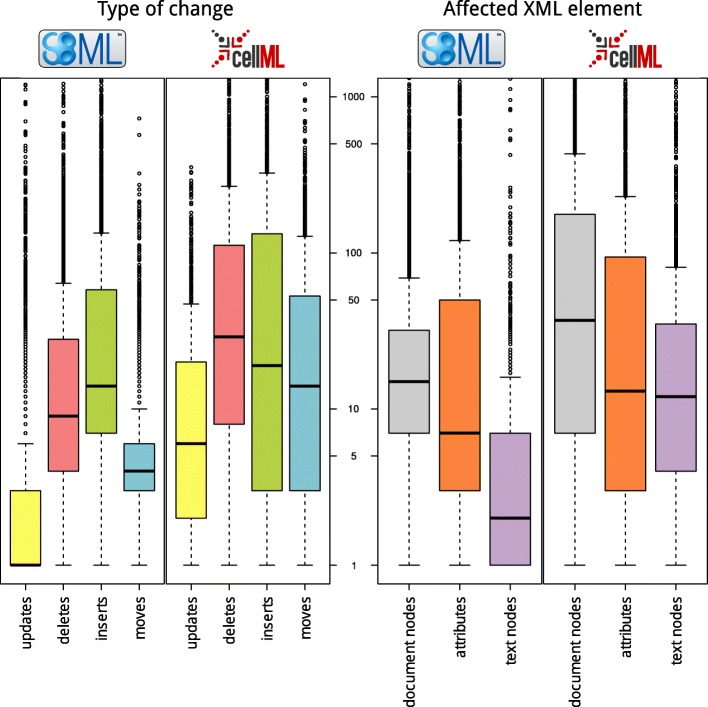
Types of diff operations. The boxplots quantify the types of changes (left boxplots) and affected components in the XML documents (right boxplots) in BioModels Database (boxplots 1 and 3) and the Physiome Model Repository (boxplots 2 and 4)

**Fig. 5:**
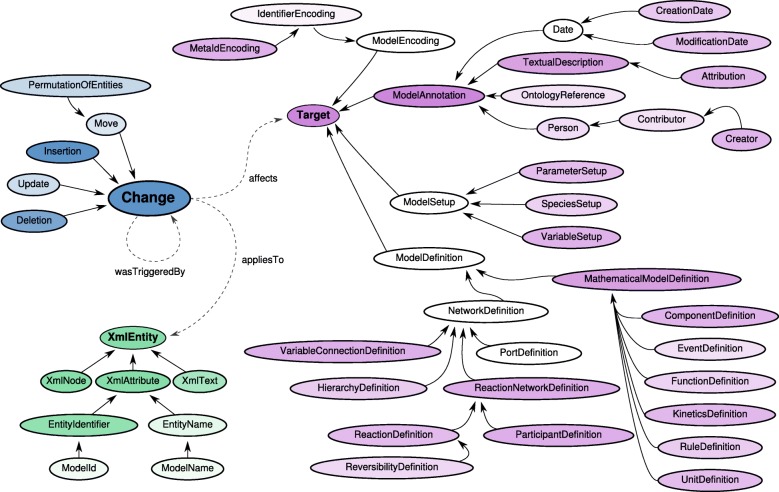
Coverage of COMODI terms. Colours are scaled individually. Thus, it is not possible to derive a quantitative statement between nodes of different colours. Only terms from the same branch can be compared

For this paper we generated static figures which provide a global view of model evolution. Figures 2, 3, and 4 were generated using aforementioned R scripts^10^. Figure 2 shows how the repositories evolve over time (number of models, size of the models). Figure 3 shows how frequently models are updated and how significant the changes are. Figure 4 shows the different types of changes and affected parts in the model document. Figure 5 was generated by an extra module implemented in the SG. It is based on an SVG template derived from the COMODI ontology [7] and visualises types and targets of changes.

### MoSt: interactive visualisations

The ModelStats website (MoSt) at most.bio.informatik.uni-rostock.de allows for interactive visualisations of the data presented here. The portal makes active use of Javascript libraries such as JQuery^11^, D3 [11], and highlight.js^12^ to provide intuitive access to the model evolution in open repositories. It integrates the DiViL^13^ tool to visualise the differences between reaction networks.

## Results

In this paper we study the evolution of SBML and CellML models from BioModels Database and the Physiome Model Repository. Overall, we analysed 3781 models, with a total of 16710 model versions. The results are obtained after applying the pipeline described in Fig. 1.

### Trends in model repositories

Figure 2 shows number and size of reusable models in the Physiome Model Repository and BioModels Database. The left panel verifies a steady increase in the number of models in both repositories.

The right panel furthermore reveals a significant increase in average number of nodes per model for BioModels Database. This observation confirms previous observations in the literature [12, 13].

The first heavy increase appears in June 2013, when the average number of nodes rises from 4866 to 13118 nodes per model. This increase is due to the publication of a large SBML model encoding the global reconstruction of human metabolism (Recon2 [14]). A second increase can be observed in February 2014, when the SBML encoding of a genome scale metabolic model was published [15]. Surprisingly, the average number of nodes remains stable for the Physiome Model Repository. As of July 2017, the average number of nodes per model is 31059.1 for BioModels Database and 863.6 for the Physiome Model Repository.

### Frequency of updates

Figure 3 visualises model updates in BioModels Database. Each coloured point indicates a change of a model in a specific release. The colour intensity reflects the number of changes: the darker the colour, the larger the number of modifications. Besides proving that models are subject to changes, the figure also reveals interesting patterns: horizontal blue bars indicate that some releases affect the majority of models. For example, the updates in December 2008 can be explained by the newly included instructions in every model on how to cite BioModels Database [16]. The blue line in May 2012 can be explained by a change in BioModels Database’s legal terms: all models were published under the terms of the *CCO Public Domain Dedication;* their *notes* section was updated accordingly.

Another set of updates relates to the SBML annotation scheme. In June 2006, the introduction of qualified references to external resources [17] affected all annotated models. In August 2012, URNs in the annotations were replaced by links to *identifiers.org*, causing another major update of the database. In June 2017, the BioModels Database team revised the annotations in a majority of their models. For example, they annotated many models with terms from GO, renamed qualifiers (e.g. bqbiol:occursIn to bqbiol:hasTaxon), and updated the timestamps of modifications.

As of July 2017, we observe an average of 4.49 versions per model in its first five years after publication. BiVeS reports an average of 327.92 between two subsequent versions of a model. However, not all changes do necessarily influence the behaviour of the model. Some are due to format updates, to design changes, or to changes in the model annotation [7].

### Delta composition and characterisation of changes

Figure 4 quantifies the delta compositions. The left-hand side shows the type of changes in SBML (boxplot 1) and CellML (boxplot 2) documents, respectively. We distinguish four types of changes: *inserts*, *deletes*, *updates*, and *moves*. The majority of changes are inserts and deletes; there are just a few updates. Tendentially, there are more differences between versions of a CellML document. More specifically, we see that entities in the CellML documents move more frequently than the ones in SBML documents.

The models considered in the study are all encoded in the Extensible Markup Language (XML). XML supports the concepts of elements (*document nodes*), attributes that further describe elements (*attributes*), and human readable pieces of text (*text nodes*). Both SBML and CellML are derivates of XML and define the basic structure of a model using *document nodes*. Attributes store further information about model entities. In SBML, for example, a biological entity is represented by a *document node* which may then contain *attributes*, such as an initialConcentration. Both formats use *text nodes* to, for example, store meta information about a model or its entities. The right-hand side of Fig. 4 shows that the least modifications in SBML documents affect the *text nodes*. Most updates in CellML documents affect *document nodes*, while the frequency of changes on *text nodes* and *attributes* are relatively similar.

The decision whether a change is relevant or not cannot always be made automatically. However, it helps to determine where a change takes effect in the model. Using the COMODI ontology, information about the characteristics can be described semantically. Our pipeline is able to annotate changes with a subset of the COMODI ontology. However, it is not able to derive information about the intention nor the reason of a change.

Figure 5 shows the branches of the COMODI ontology coloured in blue (Change), purple (Target), and green (XmlEntity). The intensity of the colour indicates how often a difference has been automatically classified with the associated term. For example, it is apparent that the terms *Insertion* and *Deletion* are darker than *Update* and *Move*, which is in concordance with Fig. 4.

### The ModelStats website

An interactive access to the data presented here is offered by the MoSt web tool (most.bio.informatik.uni-rostock.de ). MoSt provides a number of filters. It is, for example, possible to specify a time range or the model format. Furthermore, the data can be filtered for specific model identifiers. Thus, the evolution of a single model or a subset of models can be analysed. Repositories may link from a model’s page to information about its evolution in MoSt. For example, most.bio.informatik.uni-rostock.de/#m:BIOMD0000000012,v:d,d1:2006-06-01,d2:2011-04-30 shows the evolution of model BIOMD0000000012 between June 1st 2006 and April 30th 2011. Furthermore, most.bio.informatik.uni-rostock.de/#m:BIOMD,v:d filters for all models whose identifier start with BIOMD, effectively selecting all curated models from BioModels Database^14^.

MoSt features four types of visualisations. A donut chart visualises each model transition in the selected time range; the amount of changes in a version transition is reflected by the size of the donut’s slice. A heatmap provides more details about the actual numbers of *diff* operations for each transition; the height of a heat bar corresponds to the total number of changes. Both visualisations are interactive and provide access to more details on the changes between two versions of a model. The differences can be recomputed online using the BiVeS web application and the results are shown (human readable report, graphic, XML encoded differences, COMODI terms). Finally, MoSt offers two boxplot visualisations, similar to Fig. 4. One boxplot visualises the type of change (*move*, *insert*, *delete*, or *update*) of each model transition within the selected time range. The other boxplot shows which parts in the XML documents were subject to change (*text nodes*, *attributes*, or *document nodes*). Taken together, the MoSt tool allows researchers to explore the history of models in BioModels Database and the Physiome Model Repository.

## Example: changes in the repressilator model

We chose the classical example of the Repressilator [18] to showcase how a model in BioModels Database changes over time and how our tools contribute to a better understanding of these changes. The Repressilator is a synthetic model that links three transcriptional repressors to build an oscillating network. Its practical applicability has, for example, been shown for multiple organisms, including *Arabidopsis* [19] and *Escherichia coli* [20]. The model was first released in BioModels Database in September 2005^15^ and is identified by BIOMD0000000012. In total, the SBML document has been modified 21 times. The model homepage at BioModels Database^16^ already provides a textual description for many of the changes.

**Fig. 6:**
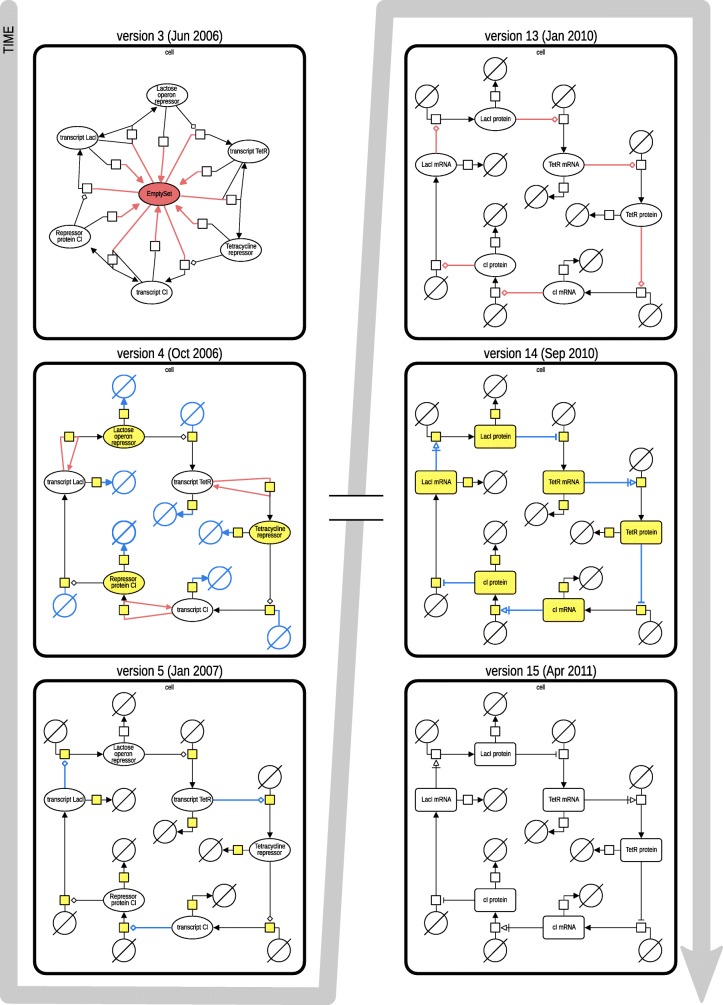
Differences between versions of the Repressilator model (BIOMD0000000012) in BioModels Database. Six versions of the Repressilator model (versions 3, 4, 5, 13, 14 and 15) are visualised in SBGN (generated using VANTED [25] and SBGN-ED [26]). The differences between the versions, as identified by BiVeS, are highlighted with a colour code: updates are in yellow, inserts are in blue and deletes are in red

Figure 6 displays six versions of the Repressilator model in SBGN (Systems Biology Graphical Notation), a standard to visualise biological networks as graphs [21]. The figure also highlights the differences between the versions as identified by BiVeS: (i) elements that have been removed in the subsequent version are coloured in red, (ii) elements that have been introduced in the current version are coloured in blue, and (iii) changes, which are not visible in the reaction network (e. g. updates of an initial concentration) are coloured in yellow. Please note that versions 3 to 5 (left column) and versions 13 to 15 (right column) are consecutive, while there is a time leap between versions 5 and 13 (left and right column).

The first transition displayed in the figure shows the deletion of one SBML entity (emptySet) between version 3 and version 4. This change is caused by a design decision on the SBML level. It does not affect the biological meaning, but it changes the SBML file and has a significant effect on the SBGN representation.

The second transition between version 4 and version 5 comprises of updates (in yellow) and changes to the reaction network (in blue and red). Specifically, the modifications rectify the effect of the transcripts over the translation of the repressors. Version 5 of the model encodes for the fact that the repressor is not created from the transcript (version 4), but that the transcripts modulate the translation of the repressors (version 5).

The third transition between version 13 and version 14 mainly improves the annotation of the model entities, reflected by a more detailed SBGN map. For example, the encoding of arrows and glyphs is more specific in version 14 (e. g., species are marked as macromolecules, *TetR protein* is described as an inhibitor for the translation of *cI mRNA*). Please note that the names of the species were updated at some point between version 5 and version 13 (not highlighted in the figure).

Finally, the fourth transition between version 14 and version 15 does not show any changes in the model. The reason for the existence of version 15 is that BioModels Database generates a new model version for every model at each release.

## Discussion

Models are continuously subjected to changes. To understand the impact, characteristics, and frequency of changes we analysed the evolution of simulation models in open model repositories. The data presented in this paper has been generated following the pipeline described in Fig. 1.

Many changes do not affect the model in the biological sense. When studying the similarity or changes in two versions of a model, different aspects may be considered [22]. Depending on the actual use of the model, some aspects are more relevant than others. In the Repressilator model, for example, the first transition (between versions 3 and 4 in Fig. 6) affects the SBML encoding of the model, but not the biological system. Similar examples are updates in the SBML specification, updated publications, or new reference schemes to external data sources. These changes, if applied to a repository, affect the majority of models (see again blue bars in Fig. 3). They typically do not affect obtained results. The knowledge about these changes can still be relevant for developers implementing tool support for SBML and CellML. However, researchers looking for changes in the biological model definition may exfiltrate the changes behind the horizontal blue bars in Fig. 3. Annotations with terms from the COMODI ontology support users in distinguishing between relevant and unimportant changes.

We also observed that some models are changed more often than others. We were not able to determine whether “famous” models are updated more frequently (e. g., because they are checked by more scientists) or less frequently (e. g., because one reason for their frequent reuse is their quality). This, however, could be an interesting endeavour for an experienced modeller. With respect to encoding formats, our data suggests that changes in CellML models are more radical in comparison to changes in SBML models, see Fig. 4. However, significant changes can be expected with the implementation of SBML Level 3 models, which have a fundamentally different structure, and may import different SBML constructs from the so-called packages [23].

The updates with release 31 of BioModels Database in June 2017 do not seem very invasive when looking at Fig. 3. However, when comparing the figure with MoSt’s filter graphic, one might speculate a discrepancy and hypothesise the large amount of changed files come from the Physiome Model Repository. This is not the case, though. The majority of the 1250 updated documents origin from BioModels Database. More specifically, 367 models from the curated branch and 640 models from the non-curated branch were affected. That means, minimal changes were introduced in about half the models from the curated branch. And indeed, the few resulting light-blue bars seem unsuspicious in Fig. 3. They are, however, very prominent in MoSt’s time-slider, which displays the number of new versions introduced at a point in time. This suggests a curation initiative at BioModels, which affected a significant amount of their models.

The figures presented in this paper are based on the state of BioModels Database and the Physiome Model Repository as of July 2017. They provide a global view on changes in models in the past years. However, during our investigation we observed that the history of model changes is not stable. It can happen that the history of releases in the repository is changed. We found two possible explanations for this. First, the Physiome Model Repository works on the basis of socalled workspaces. When a new workspace becomes public, the whole history of that workspace is also published, thereby slightly rewriting the (publicly visible) history of the whole repository. Second, we observed that the latest releases of BioModels Database can be updated up to the point when a new release is published, leading to inconsistencies in our latest data sets. Hence, it is important to remember that the figures may change in the future and yet affect shown data from the “past”.

Our investigations do not provide information about who introduced a change in a model. This information is difficult, and sometimes impossible, to retrieve. For example, in BioModels Database one cannot see who changed a file; only snapshots of the repository are openly available. We want to encourage the maintainers of repositories to provide a system where curators and modellers can transparently track the evolution of a project, e. g. using PROV-O [24] to encode the provenance and COMODI to describe reason, intention, and effects of a change. If, in future, model repositories implement such a system, incorporating that data will be an interesting extension to MoSt.

While this paper provides only a global view on available models at a certain point in time, our web portal MoSt can be used to filter the models by id, time, or format. Thus, a personalised exploration of model histories is possible. MoSt is a static web project and open source available at GitHub^17^. Thus, it is easy to create a new instance of MoSt, which allows for more control and flexibility. In addition, the generated data tables are accessible through MoSt’s web page. MoSt, in turn, is regularly being updated to reflect the latest state of BioModels Database and the Physiome Model Repository. Everyone is encouraged to extend MoSt with further useful analyses.

## Conclusions

The reuse of models is still impeded by a lack of trust and documentation. A detailed and transparent documentation of all aspects of the model, including its provenance, will improve this situation. Knowledge about a model’s provenance can avoid the repetition of mistakes that others already faced. More insights are gained into how the system evolves from initial findings to a profound understanding. It is the responsibility of the maintainers of model repositories to offer transparent model provenance to their users.

In this work, we evaluated all publicly available versions of models in BioModels Database and the Physiome Model Repository, and we searched for irregularities and interesting pattern in the plots. Our results inform scientists on how models evolve. As some changes affect the biological network, one conclusion drawn from this work is that existing models should continuously be monitored for changes. Our web tool MoSt gives access to model changes and displays the actual differences between single model versions.

## Footnotes

1
github.com/binfalse/BiVeS-StatsGenerator

2
github.com/SemsProject/ModelCrawler

3
hub.docker.com/r/binfalse/bives-statsgenerator

4
www.r-project.org

5
github.com/binfalse/BiVeS-StatsGenerator/tree/master/src/main/resources

6
most.bio.informatik.uni-rostock.de

7
github.com/SemsProject/MoSt

8
ftp://ftp.ebi.ac.uk/pub/databases/biomodels/releases/

9
models.physiomeproject.org/workspace_list_txt

10
github.com/binfalse/BiVeS-StatsGenerator/tree/master/src/main/resources

11
jquery.com

12
highlightjs.org

13
github.com/Gebbi8/DiVil

14 More information on filters and the different visualisations is available from MoSt’s project page at github.com/SemsProject/MoSt .

15
biomodels.bio.informatik.uni-rostock.de/BIOMD0000000012.xml/2005-04-11/BIOMD0000000012.xml

16 BIOMD0000000012, http://identifiers.org/biomodels.db/BIOMD0000000012

17
github.com/SemsProject/MoSt

## References

[1] King RD, Liakata M, Lu C, Oliver SG, Soldatova LN (2011). On the formalization and reuse of scientific research. J R Soc Interface.

[2] Li C, Donizelli M, Rodriguez N, Dharuri H, Endler L, Chelliah V, Li L, He E, Henry A, Stefan M, Snoep J, Hucka M, Le Novère N, Laibe C (2010). BioModels Database: An enhanced, curated and annotated resource for published quantitative kinetic models. BMC Syst Biol.

[3] Yu T, Lloyd CM, Nickerson DP, Cooling MT, Miller AK, Garny A, Terkildsen JR, Lawson J, Britten R, Hunter PJ (2011). The Physiome Model Repository 2. Bioinformatics.

[4] Hucka M, Finney A, Sauro H, Bolouri H, Doyle JC, Kitano H, Arkin AP, Bornstein B, Bray D, Cornish-Bowden A (2003). The systems biology markup language (SBML): a medium for representation and exchange of biochemical network models. Bioinformatics.

[5] Cuellar AA, Lloyd CM, Nielsen PMF, Bullivant DP, Nickerson DP, Hunter PJ (2003). An Overview of CellML 1.1, a Biological Model Description Language. Simulation.

[6] Wilkinson MD, Dumontier M, Aalbersberg IJ, Appleton G, Axton M, Baak A, Blomberg N, Boiten J-W, da Silva Santos LB, Bourne PE, et al. The FAIR Guiding Principles for scientific data management and stewardship. Sci Data. 2016;3. https://www.ncbi.nlm.nih.gov/pubmed/26978244. 10.1038/sdata.2016.18 PMC4792175 26978244

[7] Scharm M, Waltemath D, Mendes P, Wolkenhauer O (2016). COMODI: an ontology to characterise differences in versions of computational models in biology. J Biomed Semant.

[8] Waltemath D, Henkel R, Hälke R, Scharm M, Wolkenhauer O (2013). Improving the reuse of computational models through version control. Bioinformatics.

[9] Scharm M, Wolkenhauer O, Waltemath D. An algorithm to detect and communicate the differences in computational models describing biological systems. Bioinformatics. 2015;484. https://www.ncbi.nlm.nih.gov/pubmed/26490504. 10.1093/bioinformatics/btv484 PMC4743622 26490504

[10] Miller AK, Yu T, Britten R, Cooling MT, Lawson J, Cowan D, Garny A, Halstead M, Hunter PJ, Nickerson DP, Nunns G, Wimalaratne SM, Nielsen PM. Revision history aware repositories of computational models of biological systems. BMC Bioinformatics. 2011;12(1). 10.1186/1471-2105-12-22. 10.1186/1471-2105-12-22 PMC3033326 21235804

[11] Bostock M, Ogievetsky V, Heer J (2011). D ^3^ data-driven documents. IEEE transactions on visualization and computer graphics.

[12] Henkel R, Endler L, Peters A, Le Novère N, Waltemath D (2010). Ranked retrieval of computational biology models. BMC Bioinformatics.

[13] Chelliah V, Juty N, Ajmera I, Ali R, Dumousseau M, Glont M, Hucka M, Jalowicki G, Keating S, Knight-Schrijver V, et al. Biomodels: ten-year anniversary. Nucleic Acids Res. 2014;1181. https://www.ncbi.nlm.nih.gov/pmc/articles/PMC4383975/. 10.1093/nar/gku1181 PMC4383975 25414348

[14] Thiele I, Swainston N, Fleming RM, Hoppe A, Sahoo S, Aurich MK, Haraldsdottir H, Mo ML, Rolfsson O, Stobbe MD (2013). A community-driven global reconstruction of human metabolism. Nat Biotechnol.

[15] Mardinoglu A, Agren R, Kampf C, Asplund A, Nookaew I, Jacobson P, Walley AJ, Froguel P, Carlsson LM, Uhlen M (2013). Integration of clinical data with a genome-scale metabolic model of the human adipocyte. Mol Syst Biol.

[16] Le Novère N, Bornstein B, Broicher A, Courtot M, Donizelli M, Dharuri H, Li L, Sauro H, Schilstra M, Shapiro B, Snoep JL, Hucka M (2006). BioModels Database: a free, centralized database of curated, published, quantitative kinetic models of biochemical and cellular systems. Nucleic Acids Res.

[17] Le Novère N, Finney A, Hucka M, Bhalla US, Campagne F, Collado-Vides J, Crampin EJ, Halstead M, Klipp E, Mendes P (2005). Minimum information requested in the annotation of biochemical models (MIRIAM). Nat Biotechnol.

[18] Elowitz MB, Leibler S (2000). A synthetic oscillatory network of transcriptional regulators. Nature.

[19] Pokhilko A, Fernández AP, Edwards KD, Southern MM, Halliday KJ, Millar AJ. The clock gene circuit in *Arabidopsis* includes a repressilator with additional feedback loops; 8(1):574. 10.1038/msb.2012.6. 22395476. Accessed 25 July 2017. 10.1038/msb.2012.6 PMC3321525 22395476

[20] Elowitz MB, Leibler S. A synthetic oscillatory network of transcriptional regulators; 403(6767):335–8. 10.1038/35002125. Accessed 25 July 2017. 10.1038/35002125 10659856

[21] Le Novère N, Hucka M, Mi H, Moodie S, Schreiber F, Sorokin A, Demir E, Wegner K, Aladjem MI, Wimalaratne SM (2009). The systems biology graphical notation. Nat Biotechnol.

[22] Henkel R, Hoehndorf R, Kacprowski T, Knüpfer C, Liebermeister W, Waltemath D. Notions of similarity for systems biology models. Brief Bioinform. 2016;090. https://www.ncbi.nlm.nih.gov/pubmed/27742665. 10.1093/bib/bbw146 PMC5862340 28096074

[23] Hucka M, Bergmann F, Hoops S, Keating S, Sahle S, Wilkinson D, Keating SM, Wilkinson DJ. The Systems Biology Markup Language (SBML): Language Specification for Level 3 Version 1 Core (Release 1 Candidate). Nat Precedings. 2010;713. 10.1038/npre.2010.4123.1.

[24] McGuinness D, Lebo T, Sahoo S. PROV-O: The PROV Ontology. http://www.w3.org/TR/2013/REC-prov-o-20130430/. Accessed 30 Oct 2017.

[25] Rohn H, Junker A, Hartmann A, Grafahrend-Belau E, Treutler H, Klapperstück M, Czauderna T, Klukas C, Schreiber F (2012). VANTED v2: a framework for systems biology applications. BMC Syst Biol.

[26] Czauderna T, Klukas C, Schreiber F (2010). Editing, validating and translating of SBGN maps. Bioinformatics.

